# Pervasive Health Care Services and Technologies

**DOI:** 10.1155/2008/946082

**Published:** 2008-09-24

**Authors:** Sajid Hussain, Laurence T. Yang, Frédérique Laforest, Christine Verdier

**Affiliations:** ^1^Jodrey School of Computer Science, Acadia University, Wolfville, NS, Canada B4P 2R6; ^2^Department of Computer Science, St. Francis Xavier University, Antigonish, NS, Canada B2G 2W5; ^3^CNRS UMR 5205, LIRIS, INSA de Lyon, Bâtiment Blaise Pascal, 7 avenue Jean Capelle, 69621 Villeurbanne Cedex, France; ^4^CNRS UMR 5217, LIG-SIGMA, Joseph Fourier University, Bâtiment C, 220 route de la chimie, 38400 Saint-Martin-d'Hères, France

Due to recent developments in pervasive and ubiquitous computing, health care systems can provide well-informed and high-quality patient care services. A care provider can receive a simplified, adaptive, and latest view of the medical data. Although the medical file is unique, the distributed record could be accessible from any place at any time by care providers. Pervasive health care information systems allow overcoming the problem of heterogeneity of technologies and services in this domain and the papers selected for this special issue represent a good panel for addressing this challenge. Of course, the selected topics and papers are not an exhaustive representation of the area of Pervasive Health Care Services and Technologies. Nonetheless, they represent the rich and many-faceted knowledge, that we have the pleasure of sharing with the readers. We would like to thank the authors for their excellent contributions and patience in assisting us. Finally, the fundamental work of all reviewers on these papers is also very warmly acknowledged.

This special issue contains ten papers, where three papers are related to cardiac and one paper covers the identification of sleep problems. Four papers are regarding the architecture and implementation of smart homes for tele-health. Finally, two papers address security and privacy issues.

In the first paper entitled “Feasibility study and design of a wearable system-on-a-chip pulse radar for contactless cardiopulmonary monitoring,” Zito et al. present a system-on-a-chip radar sensor for next-generation wearable wireless interface for health care and safeguard. The system consists of a radar sensor for detecting the heart and breath rates and a low-power IEEE 802.15.4 ZigBee radio interface, which provides a wireless data link with remote data acquisition and control units. 

In the second paper, “Real-time and secure wireless health monitoring,” Dağtaş et al. present a framework for a wireless health monitoring system using wireless networks such as ZigBee. The proposed framework provides the detection of signals using a wireless body sensor network (BSN), low-power and reliable data transmission through ZigBee network nodes, secure transmission of medical data over BSN, efficient channel allocation for medical data transmission over wireless networks, and optimized analysis of data using an adaptive architecture that maximizes the utility of processing and computational capacity at each platform. 

In the third paper, “Development of a novel contactless mechanocardiograph device,” Tavakolian et al present a novel method of detecting mechanical movement of the heart, Mechanocardiography (MCG), with no connection to the subject's body. This measurement is based on radar technology and it has been proven through this research work that the acquired signal is highly correlated to the phonocardiograph signal and acceleration-based ballistocardiograph signal (BCG) recorded directly from the sternum. 

In the fourth paper, “PATHOS: Pervasive at home sleep monitoring,” Obermiller and Ahamed investigate home sleep monitoring. PATHOS focuses not only on analyzing patterns during the night, but also on collecting data about the subject lifestyle that is relevant and important to the diagnosis of his/her sleep. Using existing technology, PATHOS provides wireless, inexpensive, portable, and customized solution.

In the fifth paper, the research of Rammal et al entitled “An adaptive system for home monitoring using a multi-agent classification of patterns,” proposes a software architecture to monitor elderly people at home. In order to monitor efficiently the aged people, they build dynamically patterns from sensors' data and define a multi-agent method of classification used at an individual level to evaluate a risk or prevent a medical failure.

In the sixth paper entitled “Location estimation in a smart home,” Rahal et al. present the implementation of a location system of elderly people living alone at home. To do so, their system uses Bayesian filtering and a set of anonymous sensors. Their experiments have shown good accuracy and robustness.

In the seventh paper, Paganelli et al present “ERMHAN: A context-aware service platform to support continuous care networks for home-based assistance” . The main goal of this platform is to enhance appropriately information sharing between care providers in home care. They also present the architecture of their platform and preliminary experimental results. 

In the eighth paper entitled “Building application-related patient identifiers: what solution for a european country?” Quantin et al. pose the problem of a global patient identifier. In France, like in other European Countries, the patient's national identifier is unauthorized and makes the linkage between patient's data difficult. The authors propose a method using a derived social number to be used as the national identifier for patients. The solution is based on the utilization of anonymity techniques. 

In the ninth paper entitled “A Tamper-resistant and portable healthcare folder,” Anciaux et al. present the idea of a new hardware portable device to give back to the patient the control over his/her medical data. The proposed architecture assesses a secure access to data hosted both by the device and by a traditional EHR server. They also present their experiment in the context of a medico-social network for the elderly.

*Sajid Hussain* *Sajid Hussain*

*Laurence T. Yang* *Laurence T. Yang*

*Frédérique Laforest* *Frédérique Laforest*

*Christine Verdier* *Christine Verdier*

